# Clinical significance of CA-125 in elderly patients with active pulmonary tuberculosis: A retrospective study

**DOI:** 10.15537/smj.2022.43.11.20220460

**Published:** 2022-11

**Authors:** Ping Zhao, Liang Chen, Ze Q. Xie, Ji Y. Jian, Pan P. Sun

**Affiliations:** *From the Department of Clinical Laboratory (Zhao, Chen, Xie, Jian, Sun), Beijing Shijitan Hospital, Capital Medical University; and from Beijing Key Laboratory of Urinary Cellular Molecular Diagnostics (Zhao, Chen, Xie, Jian, Sun), Beijing, China*

**Keywords:** tuberculosis, pulmonary, antigen, CA-125, elderly, effusion, pleural

## Abstract

**Objectives:**

To assess the clinical significance of serum CA-125 levels in elderly patients with pulmonary tuberculosis (PTB).

**Methods:**

We retrospectively analyzed 1613 participants–patients (aged ≥60 years) admitted to the Beijing Shijitan Hospital, Beijing, China from February 2015 to January 2021 and healthy participants, divided into 4 groups: PTB (group 1), pulmonary malignancies (group 2), pulmonary non-malignant diseases (group 3), and healthy participants (group 4). Data concerning demographics, physical examination findings, computed tomography, histopathological examination, and laboratory tests for *Mycobacterium tuberculosis* and serum CA-125 levels were collected and analyzed.

**Results:**

There were 720 healthy individuals and 893 patients in the study. The median levels and abnormal rates of CA-125 in groups 1 (42.5, 57.3%) and 2 (34.4, 49.5%) were higher than those in groups 3 (21.1, 29.2%) and 4 (8.6, 0.4%) (*p*<0.05). The ordinal logistic regression analysis model revealed significant associations between CA-125 levels and PTB (OR and 95% confidence interval [CI]: 2.749 (1.876-4.027)), hypoproteinemia [OR and 95% CI: 1.519 (1.114-2.070)], serous effusion [OR and 95% CI: 7.364 (5.346-10.143)], pulmonary malignancy [OR and 95% CI: 2.206 (1.518-3.204)], respiratory failure [OR and 95% CI: 3.216 (2.087-4.956)], and cor pulmonale [OR and 95% CI: 2.990 (1.282-6.973)].

**Conclusion:**

Although elevated CA-125 levels may serve as a potential marker for diagnosing PTB in the elderly, they are affected by multiple factors, including serous effusion. Hence, caution is warranted while using this marker.

**T**uberculosis (TB) is a communicable bacterial disease that typically affects the lungs. As one of the top 10 causes of mortality in the world, it has been reported that approximately 10 million people suffered from TB in 2019 across the globe, of which 1.4 million people succumbed to the disease.^1^ While the disease can affect any age group, TB in older people is becoming a major public health and clinical concern, particularly in light of the aging global population. TB-related morbidity in older adults is higher than that in other 10-year age interval.^2^ Despite the availability of effective treatment, the proportion of elderly patients with active TB is still high and even rising in certain countries.^3,4^ Furthermore, this rise in disease incidence is disproportionately greater than the overall increase in the elderly population of the world.^3,4^ The reported incidence of TB in older people ranges from 13.2 per 100,000 people (Australia) to as high as 2194 cases per 100,000 (Cambodia) in some Asia-Pacific countries, which is several fold than that in the younger population.^4^ According to an epidemiological survey of TB in China, the incidence of TB increased with age, peaking in the 75-79 years age group at 866 cases per 100,000 people.^5,6^

Elderly patients with PTB experience atypical clinical symptoms and have a more complicated and longer disease course, a greater diversity of chest imaging findings with more extensive lesion distribution, and higher rates of misdiagnosis and missed diagnosis.^7^ Additionally, adverse reactions to anti-tubercular agents are more likely to occur in elderly PTB patients than in other age groups. Since early detection of PTB in the elderly is clinically challenging, it leads to poorer treatment outcomes and a potential increase in mortality. Therefore, rapid and accurate diagnosis is of paramount importance for treating and preventing the further spread of the disease.

Chest radiographic examination, acid-fast bacilli (AFB) staining, and *Mycobacterium* culture are the most commonly used approaches to diagnose pulmonary tuberculosis (PTB). Although clinically efficient, these methods have concerns regarding sensitivity and variability. Other more sensitive and rapid molecular detection methods require advanced laboratory equipment and are more expensive, thereby limiting their application in resource-limited regions. Notably, in some patients with PTB, AFB staining, sputum cultures, and deoxyribonucleic acid (DNA) testing for *Mycobacterium tuberculosis* (*M.tb*) may be negative or unavailable, necessitating the need for other diagnostic methods. In such patients, serological rapid diagnostic methods may be beneficial. One such accurate and rapid serological method, Interferon-Gamma Release Assay (IGRA), has been used to diagnose *M.tb* infection; however, the detection results have not been associated with treatment response and disease activity.^8,9^ Besides IGRA, CA-125, a high molecular weight glycoprotein expressed in the epithelial cells of the endometrium and fallopian tubes and mesothelial cells of the peritoneum, pericardium, and pleura, has been used as a diagnostic marker for PTB.^10^ Previous studies have reported an association between serum CA-125 and active PTB, suggesting its clinical significance in diagnosing PTB. However, other authors have expressed their concerns regarding the results being biased toward patients with serous effusions.^11^ The use of CA-125 in the diagnosis of PTB in the elderly population has been poorly studied.^11^ Therefore, we conducted this study to assess the clinical value of CA-125 levels in diagnosing elderly PTB.

## Methods

In this retrospective study, all participants were from the Beijing Shijitan Hospital, Beijing, China between February 2015 and January 2021 were enrolled using the following criteria: i) aged ≥60 years; ii) inpatients with complete clinical data available; iii) patients having active PTB as determined by at least 2 AFB-positive sputum smears, a positive *Mycobacterium* culture, or positive DNA testing for *M.tb*; iv) patients with pulmonary malignancy or non-malignant diseases diagnosed by clinical signs and symptoms, radiographic pulmonary abnormalities, and histopathology; v) healthy participants from health examination center of the Beijing Shijitan Hospital without respiratory diseases and any malignant diseases. Outpatients and patients with gynecologic conditions were excluded.

The study was carried out according to the principles of the Helsinki Declaration and was approved by the Ethics Committee of Beijing Shijitan Hospital, Beijing, China. The need for informed consent was waived off by the committee owing to the retrospective nature of the study.

All participants were classified into 4 groups: group 1–patients with newly diagnosed PTB and PTB combined with non-malignant pulmonary diseases; group 2–patients with pulmonary malignancies with or without concomitant non-malignant pulmonary diseases; group 3–patients with non-malignant pulmonary disorders other than PTB; and group 4–healthy participants. For this study, pulmonary non-malignant diseases included pneumonia, pulmonary emphysema, bronchial infection, pulmonary interstitial fibrosis, and chronic obstructive pulmonary disease (COPD). Each patient with PTB was randomly matched with 4 to 5 patients with pulmonary non-TB diseases and 4 to 5 healthy participants.

All patient data were extracted from the Hospital Information System of the Beijing Shijitan Hospital, Beijing, China. The data collection sheet included information about age, gender, current diseases, medical history, complications, and serum CA-125 levels. A value of ≥35 U/mL for serum CA-125 levels was considered abnormal.

### Statistical analysis

Datasets were analyzed by using IBM SPSS Statistics for Windows, version 22.0 (IBM Corp., Armonk, N.Y., USA). Categorical variables were expressed as frequency (n) and percentages (%), and median [25th percentile (P_25_), 75th (P_75_)] or mean±standard deviation (x±s) was used for continuous variables. CA-125 levels were divided into 4 grades: <35 U/mL, 35–69 U/mL, 70–139 U/mL, and >140 U/mL, and factors influencing CA-125 levels were identified by the ordinal logistic model.

## Results

**Figure 1:**
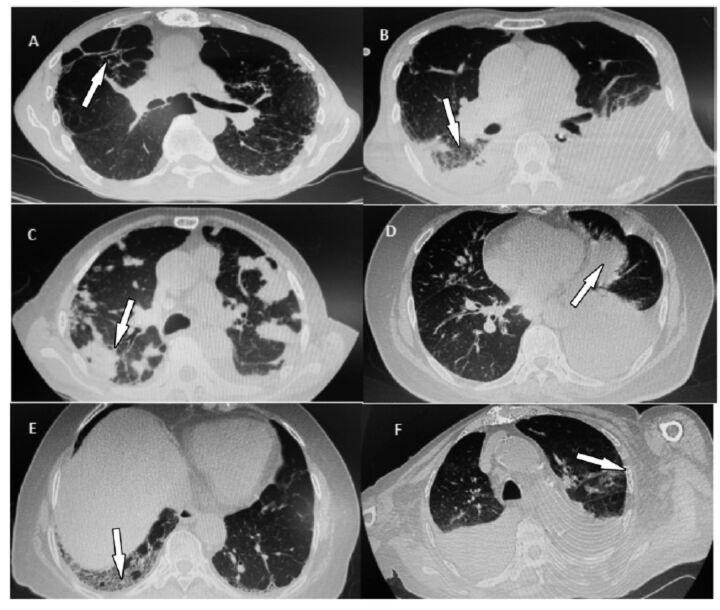
- Representative radiographic figures of patients. A) Patients form group 1, without serous effusion, AFB: 3+, serum CA-135 level; B) Patient from group 1, with serous effusion, AFB: 2+, serim CA-125 level: 250.7 U/mL; C) Patient from group 2, without serous effucion, serum CA-125 level: 186.0 U/mL; D) Patient from group 2, with serous effusion, serum CA-125 level: 195.5 U/mL; E) Patient fromo group 3, wihtout serous effusion, serum CA-125 level: 7.1 U/mL; F) Patient from gruop 3, with serous effusion, serum CA-125 level: 120.0 U/mL. AF: acid-fats bacilli.

We enrolled a total of 1613 study participants, which included 720 healthy individuals (group 4) and 893 patients (groups 1 through 3). Group 1 included 20 patients with PTB and 137 patients with PTB complicated with non-malignant pulmonary diseases. Group 2 included 113 patients with pulmonary malignancies and 196 patients with pulmonary malignancies combined with non-malignant pulmonary diseases. Group 3 included 415 patients with non-malignant pulmonary diseases. In addition to groups 1, 2, and 3, two cases of PTB combined with pulmonary malignancies and ten cases of PTB complicated with pulmonary malignancies and non-malignant diseases were also enrolled in multivariate analysis. Figure 1 presents typical radiographic features of patients belonging to groups 1 to 3.

### Group-wise comparison of demographic characteristics and CA-125 levels

In all participants (in groups 1 to 4), the differences in gender, age, and levels and abnormal rate of serum CA-125 among the 4 groups were statistically significant (Table 1).

**Table 1:** - Comparison of demographic characteristics and serum CA-125 levels among the 4 study groups.

Group	Gender (n)		Age (x±s)	CA-125 [Median (P_25_, P_75_)]	Abnormal rate of CA-125 (%)
	Male	Female			
Group 1	101	56	76.8±10.5	42.5 (21.0, 86.2)	57.3
Group 2	204	105	69.1±8.2	34.4 (14.0, 105.4)	49.5
Group 3	234	181	77.4±10.1	21.1 (12.7, 40.5)	29.2
Group 4	445	275	69.0±8.1	8.6 (1.2, 12.1)	0.4
Total test statistic	7.806		104.106	735.183	444.372
Total *p*-value	0.05		<0.001	<0.001	<0.001
Group 1 versus group 2	χ^2^=0.131, *p*=0.717		t=7.670, p *p* <0.001	H=101.997, *p*=0.146	χ^2^=2.545, *p*=0.111
Group 1 versus group 3	χ^2^=2.964, *p*=0.085		t=0.625, *p*=0.454	H=223.156, *p*<0.001	χ^2^=38.822, *p*<0.001
Group 1 versus group 4	χ^2^=0.350, *p*=0.554		t=7.778, *p*<0.001	H=757.531, *p*<0.001	χ^2^=440.334, *p*<0.001
Group 2 versus group 3	χ^2^=6.879, *p*=0.009		t=8.295, *p*<0.001	H=121.160, *p*=0.003	χ^2^=31.207, *p*<0.001
Group 2 versus group 4	χ^2^=1.648, *p*=0.199		t=0.109, *p*=0.858	H=655.535, *p*<0.001	χ^2^=405.222, *p*<0.001
Group 3 versus group 4	χ^2^=3.218, *p*=0.073		t=8.403, *p*<0.001	H=534.375, p<0.001	χ^2^=223.448, *p*<0.001

P_25_, the 25th percentile; P_75_, the 75th percentile.

Additionally, the patients were further divided into groups based on the presence or absence of serous effusion along with the disease. In participants with serous effusion (in groups 1 to 3), the differences in gender, age, and levels and abnormal rate of serum CA-125 among the 3 groups were statistically significant (Table 2).

**Table 2:** - Comparison of gender, age, and levels and abnormal rates of serum CA-125 among the four groups with serous effusion.

Group	Gender (n)		Age (x±s)	CA-125[Median (P_25_, P_75_)]	Abnormal rate of CA-125 (%)
	Male	Female			
Group 1	43	12	78.7±10.2	71.2 (38.8, 173.9)	81.8
Group 2	73	55	71.0±8.9	99.8 (44.1, 175.4)	79.7
Group 3	56	47	80.9±9.8	52.0 (24.7, 107.4)	62.1
Total test statistic	9.415		34.109	13.182	11.364
Total *p*-value	0.009		<0.001	0.001	0.003
Group 1 versus group 2	χ^2^=7.415, *p*=0.006		t=7.717, *p*<0.001	H=14.551, *p*=0.826	χ^2^=0.111, *p*=0.740
Group 1 versus group 3	χ^2^=8.689, *p*=0.003		t=2.233, *p*=0.159	H=25.100, *p*=0.208	χ^2^=6.492, *p*=0.011
Group 2 versus group 3	χ^2^=0.164, *p*=0.685		t=9.950, *p*<0.001	H=39.651, *p*=0.001	χ^2^=8.695, *p*=0.003

P_25_, the 25th percentile; P_75_, the 75th percentile.

Likewise, in participants without serous effusion (in groups 1 to 4), the differences in gender, age, and levels and abnormal rate of serum CA-125 among the 4 groups were statistically significant (Table 3).

**Table 3:** - Comparison of gender, age, and levels and abnormal rates of serum CA-125 among the four groups without serous effusion.

Group	Gender (n)		Age (x±s)	CA-125[Median (P_25_, P_75_)]	Abnormal rate of CA-125 (%)
	Male	Female			
Group 1	58	44	75.8±10.5	31.2 (18.1-68.3)	44.1
Group 2	131	50	67.8±7.3	18.0 (10.7-40.0)	28.2
Group 3	178	134	76.3±10.0	17.6 (11.3-30.1)	18.3
Group 4	445	275	69.0±8.1	8.6 (1.2, 12.1)	0.4
Total test statistic	12.601		69.316	487.826	250.03
Total *p*-value	0.006		<0.001	<0.001	<0.001
Group 1 versus group 2	χ^2^=7.077, *p*=0.008		t=7.956, *p*<0.001	H=183.969, *p*=0.001	χ2=7.396, *p*=0.007
Group 1 versus group 3	χ^2^=0.001, *p*=0.973		t=0.491, *p*=0.619	H=198.197, *p*<0.001	χ2=27.661, *p*<0.001
Group 1 versus group 4	χ^2^=0.919, *p*=0.338		t=6.754, *p*<0.001	H=613.759, *p*<0.001	χ2=310.316, *p*<0.001
Group 2 versus group 3	χ^2^=11.499, *p*=0.001		t=8.447, *p*<0.001	H=14.228, *p*=1.000	χ2=6.572, *p*=0.010
Group 2 versus group 4	χ^2^=7.008, *p*=0.008		t=1.202, *p*=0.096	H=429.790, *p*<0.001	χ2=197.834, *p*<0.001
Group 3 versus group 4	χ^2^=2.056, *p*=0.152		t=7.245, *p*<0.001	H=415.562, *p*<0.001	χ2=126.692, *p*<0.001

P_25_, the 25th percentile; P_75_, the 75th percentile.

### Factors influencing the CA-125 levels

The association between serum CA-125 levels and patients’ gender, age, diagnoses, and complications were analyzed using an ordinal logistic regression model; the factors and assignment are shown in Table 4. There was no collinearity among the variables (the tolerance of each variable >0.1, variance inflation factor <5), and the test of parallel lines revealed that the regression equations were parallel to each other (χ^2^=50.411, *p*=0.537), so the present data could be analyzed by ordinal logistic regression. Based on this, we found that significant associations exist between serum CA-125 levels and PTB, hypoproteinemia, serous effusion, pulmonary malignancy, respiratory failure, and cor pulmonale (Table 5).

**Table 4:** - Assignment for CA-125 levels and risk factors.

Factors	n	Assignment	Factors	n	Assignment
*CA-125 (U/ml)*			Liver non-malignant diseases	227	Yes = 0
					No = 1
<35	522	0	Hypoproteinemia	419	Yes = 0
					No = 1
35-	145	1	Hyperlipidemia	297	Yes = 0
					No = 1
70-	122	2	Pleural lesions	121	Yes = 0
					No = 1
140-	104	3	Coronary heart disease	399	Yes = 0
					No = 1
*Gender*			Heart failure	32	Yes = 0
					No = 1
Male	547	0	Serous effusion	290	Yes = 0
					No = 1
Female	346	1	Pulmonary emphysema	168	Yes = 0
					No = 1
*Age (year)*			Renal cyst/renal calculus	165	Yes = 0
					No = 1
60-	356	0	Bronchial diseases	350	Yes = 0
					No = 1
70-	214	1	Pulmonary interstitial fibrosis	150	Yes = 0
					No = 1
80-	255	2	COPD	141	Yes = 0
					No = 1
90-	68	3	Pulmonary malignancies	321	Yes = 0
					No = 1
PTB	169	Yes = 0	History of other malignant tumors	57	Yes = 0
		No = 1			No = 1
Obsolete PTB	70	Yes = 0	Respiratory failure	155	Yes = 0
		No = 1			No = 1
Pneumonia	570	Yes = 0	Pyohemia	87	Yes = 0
		No = 1			No = 1
Diabetes mellitus	266	Yes = 0	Cor pulmonale	23	Yes = 0
		No = 1			No = 1
Arteriosclerosis	334	Yes = 0			
		No = 1			

PTB: pulmonary tuberculosis, COPD: chronic obstructive pulmonary disease

**Table 5:** - Factors influencing serum CA-125 levels in elderly patients.

Factors	β	SE	Wald χ2	*P*-value	OR	95% CI for OR	
						Lower	Upper
PTB	1.011	0.1949	26.925	<0.001	2.749	1.876	4.027
Hypoproteinemia	0.418	0.1580	6.997	0.008	1.519	1.114	2.070
Serous effusion	1.997	0.1634	149.340	<0.001	7.364	5.346	10.143
Pulmonary malignancy	0.791	0.1905	17.244	<0.001	2.206	1.518	3.204
Respiratory failure	1.168	0.2206	28.028	<0.001	3.216	2.087	4.956
Cor pulmonale	1.095	0.4320	6.428	0.011	2.990	1.282	6.973

PTB: pulmonary tuberculosis, β: regression coefficient, SE: standard error;, OR: odds ratio, CI: confidence interval

## Discussion

Typically, the presence of *M.tb* or its DNA in respiratory samples is used to confirm the diagnosis of PTB. However, in some patients, *M.tb* may not be detectable in the respiratory sample, or a sample may not be available; in these patients, serological tests, such as the detection of CA-125, can be used to assist the diagnostic process.

Owing to the physiological aging of different organs and a concomitant decline in immunity, PTB in the elderly is associated with several complications and atypical symptoms.^7^ The primary clinical symptoms and signs of PTB in elderly patients include cough, expectoration, hemoptysis, weight loss, night sweats, fatigue, dyspnea, and chest pain.^4,12^ However, these clinical manifestations are non-specific, and a similar clinical picture may be encountered in pulmonary malignancies or non-malignant pulmonary diseases other than PTB. Furthermore, the chest imaging findings in the elderly may not be typical of PTB in terms of the location and pattern of the lesions, and the sensitivity of bacteriological examination is low and variable.^13-15^ Notably, it is also difficult for elderly PTB patients to produce high-quality respiratory specimens for bacteriological detection. Previous studies have stated that, in the elderly, the sensitivity of tuberculin skin test (TST) in diagnosing latent TB infection (LTBI) is lower, and there are more indeterminate results for IGRA in diagnosing LTBI.^13-16^ In addition, the positivity rate of IGRA in the diagnosis of TB is lower in older adults than in young people, and the sensitivity decreases with age.^17,18^

In the present study, we assessed the clinical value of serum CA-125 levels in elderly PTB patients (≥60 years old). We found that the median levels and abnormal rates of serum CA-125 were higher in PTB patients and in patients with pulmonary malignancies than in patients with non-malignant pulmonary disorders and in healthy participants (*p*<0.05). However, the differences in level and abnormal rate of serum CA-125 between PTB patients and patients with pulmonary malignancies were not statistically significant (*p*>0.05). We also found that in participants with serous effusion, the median level and abnormal rate of serum CA-125 were higher in patients with pulmonary malignancies than in patients with non-malignant pulmonary disorders (*p*<0.05). However, the differences in serum CA-125 levels between PTB patients and patients with pulmonary malignancies, and between PTB patients and patients with non-malignant pulmonary disorders were not statistically significant (*p*>0.05). The abnormal rate of serum CA-125 in PTB patients was also higher than that in patients with non-malignant pulmonary disorders (*p*<0.05); likewise, the difference between PTB patients and patients with pulmonary malignancies was also not statistically significant (*p*>0.05). In participants without serous effusion, the median level and abnormal rate of serum CA-125 were also higher in PTB patients than in the other 3 groups (*p*<0.05). These results indicate that serum CA-125 levels were elevated in elderly patients with PTB, and CA-125 test may be beneficial for diagnosing elderly PTB; however, accurate detection may be influenced by serous effusion. In addition, using different classifications (study cohort as a whole, with serous effusion, and without serous effusion), the differences in gender and age were statistically significant among all groups, which seemed to influence the CA-125 levels. However, our subsequent multivariate analysis suggested that gender and age were not the influencing factors for elevated CA-125 levels.

CA-125 was initially identified by molecular cloning as a high molecular weight glycoprotein that promotes ovarian cancer cell growth and is also present in the epithelium of tracheal, bronchial, bronchiolar, terminal bronchioles, in the glands of trachea and bronchi, and the mesothelium of pleura.^19,20^ Previous studies have highlighted the potential role of CA-125 levels in diagnosing PTB and monitoring the therapeutic efficacy of anti-TB treatment; notably, these levels were also associated with the severity of PTB.^10,11,21^ A possible explanation for these high levels of CA-125 in PTB patients is that PTB is associated with the destruction of bronchial epithelial cells, which induces increased CA-125 levels in these patients. However, some studies also reported a lower proportion (38%-48.7%) of PTB patients with high levels of CA-125, indicating that the levels of CA-125 may not necessarily be elevated in most PTB patients.^11^ Du et al^10^ immunohistochemically stained CA-125 in 6 tissue specimens from patients with active PTB and revealed that 3 specimens (50%) were positive for CA-125. In our study, 81.8% of PTB patients with serous effusion had abnormal CA-125 levels, while only 44.1% of PTB patients without serous effusion had elevated levels. Therefore, caution is warranted when applying CA-125 detection methods for diagnosing PTB in the elderly.

In addition to the significant effect of serous effusion and PTB on the CA-125 levels, we also found that hypoproteinemia, pulmonary malignancies, respiratory failure, and cor pulmonale may elevate the levels of CA-125 in elderly patients; however, age and gender did not influence these levels. Since CA-125 is expressed in the mesothelial cells of the peritoneum and pericardium, increased CA-125 levels can also be observed in diseases involving the gastrointestinal and circulatory systems. Therefore, serous effusions from those structures may have led to elevated CA-125 levels.^11,22,23^ Adenocarcinomas and diabetes mellitus may also elevate CA-125 levels.^10^ In this study, pulmonary malignancies were found to elevate serum CA-125 levels, but no such association was seen in diabetic patients. Further research exploring the association between hypoproteinemia, respiratory failure, cor pulmonale, and CA-125 is needed.

### Study limitations

Since the study was a retrospective analysis, it may suffer from selection bias. The levels of CA-125 were not monitored during and after treating patients with PTB or pulmonary malignancies, which may weaken the said association between CA-125 and PTB. Lastly, there may be other factors affecting CA-125 levels that were not included in the analysis such as multiple lobar lesions.^10^

In conclusion, the findings confirm that the levels and abnormal rates of CA-125 in elderly people with PTB are significantly higher than those observed in elderly people with other pulmonary diseases. These results highlight the use of serum CA-125 in diagnosing PTB in older adults and that the combined application of serum CA-125 and IGRA may further improve diagnostic efficiency. However, the rates of abnormal CA-125 levels were not as high in elderly PTB patients without serous effusions as anticipated (only 44.1%) since CA-125 levels can be influenced by many factors. Therefore, despite its potential clinical significance in the diagnosis of PTB in the elderly, it is necessary to be cautious when incorporating CA-125 levels into clinical practice.
